# Pancreatic T2* Magnetic Resonance Imaging for Prediction of Cardiac Arrhythmias in Transfusion-Dependent Thalassemia

**DOI:** 10.3390/jcm12186015

**Published:** 2023-09-16

**Authors:** Antonella Meloni, Laura Pistoia, Paolo Ricchi, Vincenzo Positano, Filomena Longo, Zelia Borsellino, Valerio Cecinati, Giuseppe Messina, Elisabetta Corigliano, Rosamaria Rosso, Riccardo Righi, Giuseppe Peritore, Stefania Renne, Antonino Vallone, Filippo Cademartiri

**Affiliations:** 1 Department of Radiology, Fondazione Gabriele Monasterio CNR-Regione Toscana, 56124 Pisa, Italy; antonella.meloni@ftgm.it (A.M.); laura.pistoia@ftgm.it (L.P.); positano@ftgm.it (V.P.); 2 Unità Operativa Complessa Bioingegneria, Fondazione Gabriele Monasterio CNR-Regione Toscana, 56124 Pisa, Italy; 3 Unità Operativa Semplice a Valenza Dipartimentale Ricerca Clinica, Fondazione Gabriele Monasterio CNR-Regione Toscana, 56124 Pisa, Italy; 4 Unità Operativa Semplice Dipartimentale Malattie Rare del Globulo Rosso, Azienda Ospedaliera di Rilievo Nazionale “Antonio Cardarelli”, 80131 Napoli, Italy; paolo.ricchi@aocardarelli.it; 5 Unità Operativa Day Hospital della Talassemia e delle Emoglobinopatie, Azienda Ospedaliero-Universitaria “S. Anna”, 44124 Cona, Italy; filomena.longo@ospfe.it; 6 Unità Operativa Complessa Ematologia con Talassemia, ARNAS Civico “Benfratelli-Di Cristina”, 90134 Palermo, Italy; zelia.borsellino@arnascivico.it; 7 Struttura Semplice di Microcitemia, Ospedale “Santissima Annunziata”, 74123 Taranto, Italy; valerio.cecinati@als.taranto.it; 8 Centro Microcitemie, Grande Ospedale Metropolitano “Bianchi-Melacrino-Morelli”, 89100 Reggio Calabria, Italy; gspmessina@virgilio.it; 9 Ematologia Microcitemia, Ospedale San Giovanni di Dio—ASP Crotone, 88900 Crotone, Italy; krthal@libero.it; 10 Unità Operativa Talassemie ed Emoglobinopatie, Azienda Ospedaliero-Universitaria Policlinico “Vittorio Emanuele”, 95100 Catania, Italy; rosellinarosso@gmail.com; 11 Diagnostica per Immagini e Radiologia Interventistica, Ospedale del Delta, 44023 Lagosanto, Italy; riccardo.righi@ausl.fe.it; 12 Unità Operativa Complessa di Radiologia, ARNAS Civico “Benfratelli-Di Cristina”, 90127 Palermo, Italy; giuseppe.peritore@hotmail.it; 13 Struttura Complessa di Cardioradiologia-UTIC, Presidio Ospedaliero “Giovanni Paolo II”, 88046 Lamezia Terme, Italy; stefania.renne@virgilio.it; 14 Reparto di Radiologia, Azienda Ospedaliera “Garibaldi” Presidio Ospedaliero Nesima, 95126 Catania, Italy; ninovallone@hotmail.com

**Keywords:** transfusion-dependent thalassemia, pancreas T2*, magnetic resonance imaging, prognosis, cardiac complications

## Abstract

We assessed the value of pancreatic T2* magnetic resonance imaging (MRI) for predicting cardiac events from a large prospective database of transfusion-dependent thalassemia (TDT) patients. We considered 813 TDT patients (36.47 ± 10.71 years, 54.6% females) enrolled in the Extension-Myocardial Iron Overload in Thalassemia Network. MRI was used to measure hepatic, pancreatic, and cardiac iron overload (IO), to assess biventricular function and atrial dimensions, and to detect replacement myocardial fibrosis. The mean follow-up was 50.51 ± 19.75 months. Cardiac complications were recorded in 21 (2.6%) patients: one with heart failure (HF) and 20 with arrhythmias. The single patient who developed HF had, at the baseline MRI, a reduced pancreas T2*. Out of the 20 recorded arrhythmias, 17 were supraventricular. Pancreatic T2* values were a significant predictor of future arrhythmia-related events (hazard ratio = 0.89; *p* = 0.015). Pancreas T2* remained significantly associated with future arrhythmias after adjusting for any other univariate predictor (age and male sex, diabetes, history of previous arrhythmias, or left atrial area index). According to the receiver-operating characteristic curve analysis for arrhythmias, a pancreas T2* < 6.73 ms was the optimal cut-off value. In TDT, pancreatic iron levels had significant prognostic power for arrhythmias. Regular monitoring and the development of targeted interventions to manage pancreatic IO may help improve patient outcomes.

## 1. Introduction

Beta-thalassemia is a common hereditary blood disease that affects the production of the beta-globin chain, leading to ineffective erythropoiesis, chronic hemolysis, and anemia [1,2]. Transfusion-dependent thalassemia (TDT) is the most severe clinical form of β-thalassemia, typically encompassing individuals with the thalassemia major (TM) phenotype, and is characterized by early presentation to clinical care due to severe anemia [3]. The introduction of regular red blood cell transfusions around 60 years ago has been a transformative milestone in the management of TDT, turning a once-fatal childhood disorder into a manageable chronic condition. The main objective of the lifelong tailored transfusion program is to raise the hemoglobin level to a target range of 9–10 g/dL before the next scheduled transfusion [4,5].

However, because the body lacks efficient mechanisms to eliminate excess iron [6,7], a major challenge associated with regular transfusions is the risk of secondary iron overload. Iron overload can lead to multiorgan complications, including heart disease, liver dysfunction, endocrine disorders, and other related health problems [8,9,10,11,12,13,14,15]. The combination of chelation therapy, aimed at reducing or preventing iron stores, and advanced imaging techniques like T2* magnetic resonance imaging (MRI), has revolutionized the management of iron overload in thalassemia, significantly improving the survival rate and overall outcomes [16,17,18]. The T2* MRI technique has been established as a valid means for the noninvasive assessment of organ-specific iron overload, enabling the development and the regular monitoring of personalized chelation therapy plans [19,20,21,22,23,24,25]. In particular, the aggressive responses to the presence of myocardial iron overload (MIO) guided by the T2* MRI technique have played a significant role in reducing the incidence of heart failure (HF) and heart failure-related mortality in TDT [17,18,26,27].

In recent years, with the widespread use of T2* cardiac magnetic resonance (CMR), it has become evident that arrhythmias in TDT patients are mostly of supraventricular origin and that MIO is less strongly associated with the development of arrhythmias compared to heart failure [28,29,30]. A study exploiting the full multiparametric potential of CMR beyond MIO assessment has highlighted the importance of left atrial dilatation as an independent prognosticator of supraventricular arrhythmias [30]. Moreover, diabetes mellitus (DM) emerged as a significant univariate prognosticator of arrhythmias [30]. The pathogenesis of DM in TDT involves multiple factors, and pancreatic iron overload, leading to impaired insulin release and subsequent insulin deficiency, is a significant contributor [31]. Indeed, a significant association between pancreatic iron levels quantified by T2* MRI and alterations in glucose metabolism has been demonstrated [12,15]. Moreover, pancreatic T2* values exhibited a significant correlation with cardiac T2* values [15,32,33,34,35], likely attributable to the presence of the same L-type calcium iron channels in the two organs [36]. Interestingly, a cross-sectional study involving a large cohort of TDT patients showed that arrhythmias, as well as heart failure, were associated with significantly lower pancreatic T2* values, suggesting a profound link between pancreatic iron and heart disease [15].

To our current knowledge, no study has evaluated the prognostic role of pancreatic iron in TDT patients. We postulated that the measurement of pancreatic iron burden may play a role in cardiovascular risk stratification and should therefore be taken into account to better tailor iron chelation to the needs of patients.

The goal of this multicenter study was to determine the value of pancreatic T2* MRI for predicting cardiac events (heart failure and arrhythmias) from a large prospective database of TDT patients who started regular transfusions since early childhood.

## 2. Materials and Methods

### 2.1. Patients

We considered 836 TDT patients (36.68 ± 10.69 years; 455 females). All patients were white, regularly transfused since early childhood to maintain pretransfusion hemoglobin > 9–10 g/dL, and chelated. The mean age at the start of regular transfusions was 18.01 ± 39.87 months, while the age at the start of chelation treatment was 4.94 ± 5.89 years.

The patients performed their first T2* MRI for pancreatic iron overload assessment within the Extension-Myocardial Iron Overload in Thalassemia (E-MIOT) project between October 2015 and January 2022. The E-MIOT project is an Italian network composed of 66 thalassemia centers and 13 MRI sites where a homogeneous, standardized, and validated T2* MRI technique is adopted [37]. All centers are linked by a web-based database that collects the clinical-anamnestic history of the patients, from birth to the date of the first MRI scan. The clinical, instrumental, and laboratory data are updated at every MRI of control, performed, according to the study design, every 18 ± 3 months.

The study complied with the Declaration of Helsinki and was approved by the institutional ethics committee. All patients provided written informed consent.

### 2.2. Magnetic Resonance Imaging

All patients underwent MRI using conventional clinical 1.5 T scanners from three main vendors (GE Healthcare, Milwaukee, WI, USA; Philips Healthcare, Best, Netherlands; and Siemens Healthineers, Erlangen, Germany), equipped with a phased-array receiver surface coil.

For the quantification of tissue iron levels, T2* multiecho gradient-echo sequences were acquired. Five or more axial slices, including the whole pancreas [38], a mid-transverse hepatic slice [39], and three parallel short-axis views (basal, medium, and apical) of the left ventricle (LV) [18] were obtained. T2* images were analyzed by trained radiologists (>ten years of experience) using a custom-written, previously validated software (HIPPO MIOT^®^, Version 2.0, Consiglio Nazionale delle Ricerche and Fondazione Toscana Gabriele Monasterio, Pisa, Italy, Year 2015). Three small regions of interest (ROIs) were manually delineated over the pancreatic head, body, and tail, encompassing parenchymal tissue and bypassing large blood vessels or ducts and regions with susceptibility artifacts caused by gastric or colic intraluminal gas [38]. The global pancreatic T2* value was obtained by averaging the T2* values from the three regions. Hepatic T2* values were quantified in a circular ROI of fixed dimension [39] and converted into liver iron concentration (LIC) by applying the Wood’s calibration curve [40]. The LV was divided into 16 segments, according to the standard AHA/ACC model [41], and the T2* value was obtained for all segments [18]. The global heart T2* value was derived as the average of segmental values.

Steady-state free procession (SSFP) cine images were acquired in sequential 8-mm short-axis slices from the atrioventricular ring to the apex to assess biventricular size and function in a standard and reproducible way [28]. Areas of the left and right atria were measured from the four-chamber view projection in the ventricular end-systolic phase. Biventricular volumes, LV mass, and biatrial areas were normalized by body surface area (BSA).

To determine the presence of focal/macroscopic myocardial fibrosis, late gadolinium enhancement (LGE) short-axis and vertical, horizontal, and oblique long-axis images were acquired 10–18 min after Gadobutrol (Gadovist^®^; Bayer Schering Pharma; Berlin, Germany) intravenous administration at the standard dose of 0.2 mmol/kg. LGE images were not acquired in patients presenting with a glomerular filtration rate < 30 mL/min/1.73 m^2^, refusing the contrast medium administration, or with less than 10 years of age. LGE was deemed to be present when observed in two different views [30].

### 2.3. Diagnostic Criteria and Clinical Follow-Up

HF was diagnosed by clinicians based on symptoms, signs, and instrumental findings, in accordance with the AHA/ACC guidelines [42]. Arrhythmias were diagnosed if documented by ECG or 24-h Holter ECG and if requiring specific medications. The classification of arrhythmias was performed according to the AHA/ACC guidelines [43]. The term “cardiac complications” included HF and arrhythmias.

The end of the follow-up coincided with the date of the last available MRI. For patients who did not undergo a control MRI, a case report form detailing patient outcomes between the baseline MRI and April 2023 was completed by the caring hematologist. If a patient developed more than one complication during the follow-up, only the first one was taken into account.

### 2.4. Statistical Analysis

All data were analyzed using SPSS version 27.0 (IBM Corp., Armonk, NY, USA) and MedCalc version 19.8 (MedCalc Software Ltd., Ostend, Belgium) statistical packages.

Continuous variables were described as mean ± standard deviation (SD). Categorical variables were expressed as frequencies and percentages. The normality of the distribution of the continuous variables was assessed using the Kolmogorov–Smirnov test.

For continuous variables that followed a normal distribution, comparisons between two groups were conducted using an independent-samples *t*-test. Levene’s test was employed to verify the homogeneity of variances (homoscedasticity). In cases where Levene’s test yielded a significance level of less than 0.05, indicating that homoscedasticity could not be assumed, the Welch statistic was used. The Wilcoxon signed-rank test was employed for continuous values that did not exhibit a normal distribution. χ^2^ testing was conducted for categorical variables.

Analysis of covariance (ANCOVA) models were used to assess how potential covariates influenced the differences between groups concerning clinical and MRI parameters. Covariates were integrated into the analysis if a variable showed a significant difference between the groups and an association with the assessed outcome. When necessary, outcomes were log-transformed to achieve normality in the residual distributions and to equalize the residual variance.

Correlation analysis was conducted using Pearson’s or Spearman’s tests, where appropriate.

The Cox proportional hazard model was employed to examine the association between the prognostic variables under consideration and the outcome. The results were reported as hazard ratios (HRs) with 95% confidence intervals (CI).

Kaplan–Meier curves were generated by relating the development of an outcome over time to each significant prognosticator. The log-rank test was employed to compare different strata in Kaplan–Meier analyses.

To identify the optimal pancreas T2* cut-off for distinguishing the presence of a specified outcome, the maximum sum of sensitivity and specificity was computed from receiver-operating characteristic (ROC) curve analysis.

In all tests, a 2-tailed *p* < 0.05 was considered statistically significant.

## 3. Results

### 3.1. Baseline Findings

Twenty-three patients were excluded from this study due to the presence of a cardiac complication (16 arrhythmias, 5 HF, 2 HF + arrhythmias) at the baseline MRI.

The baseline demographic, clinical, and MRI features of the 813 considered TDT patients are summarized in Table 1.

Patients were homogeneously distributed in terms of gender, and the mean age was 36.47 ± 10.71 years. Mean global pancreas T2* was 13.16 ± 10.49 ms, and global pancreas T2* was significantly lower in patients with diabetes than in those without diabetes (8.09 ± 5.09 ms vs. 13.85 ± 10.86 ms; *p* < 0.0001).

The contrast medium was administered only to 224 (27.6%) patients.

All patients were naïve to pancreatic T2* MRI, while 671 (82.5%) of them had already undergone at least one MRI scan for the quantification of cardiac and hepatic iron levels. These patients had been previously enrolled in the MIOT project, which lasted from 2006 to 2015 [27].

### 3.2. Cardiac Complications

The mean follow-up time was 50.51 ± 19.75 months (median = 51.94 months). Cardiac complications were recorded in 21 (2.6%) patients: one with HF and 20 with arrhythmias.

The single patient who developed heart failure (six months after the MRI) was a female with diabetes and a history of ischemic stroke. At the baseline MRI, she had a global heart T2* value of 47.50 ms and a reduced pancreas T2* (5.50 ms).

Out of the 20 recorded arrhythmias, three were ventricular and 17 were supraventricular (15 atrial fibrillation and two atrial flutter). The mean time from the MRI to an arrhythmia episode was 30.28 ± 20.53 months (range: 1–67 months).

In light of the increased prevalence of arrhythmias, all analyses were conducted considering only this cardiac disease.

### 3.3. Characterization of Patients Who Developed Cardiac Arrhythmias

Table 1 shows the comparison of baseline demographic and MRI parameters between patients who experienced arrhythmias and those who remained free of arrhythmias. Patients with arrhythmias were significantly older and more frequently males. No significant difference was detected in the frequency of splenectomy or baseline pretransfusion hemoglobin and serum ferritin levels, but arrhythmias were associated with an increased frequency of diabetes mellitus. Baseline hepatic and cardiac iron levels were comparable between the two groups, while baseline pancreas T2* values were significantly lower in patients who developed arrhythmias compared to those who remained free of arrhythmias.

Age exhibited a significant inverse correlation with global pancreas T2* values (R = −0.219; *p* < 0.0001) and was therefore used as a covariate in the ANCOVA. The difference in global pancreas T2* values remained significant even after the adjustment for age (*p* = 0.043). Compared to patients who were arrhythmia-free, patients with arrhythmias had comparable biventricular function parameters and prevalence of replacement myocardial fibrosis at baseline, but they exhibited significantly higher atrial areas indexed by BSA. Biatrial areas were independent of age, so ANCOVA correction was not performed.

### 3.4. Prognostic Markers of Arrhythmias

Table 2 shows the results of the univariate Cox regression analysis for the prediction of arrhythmias. Among the non-MRI parameters, aging, male sex, and diabetes emerged as significant univariate prognosticators. Among the MRI parameters, baseline global pancreas T2* and both atrial areas emerged as univariate prognosticators. In a multivariate model including both atrial areas, the left atrial area index remained the only independent predictor.

Thirteen (1.6%) patients had a prior and resolved history of arrhythmias, all supraventricular (12 atrial fibrillation and one atrial flutter). Patients who developed arrhythmias during the FU had a more frequent positive history of past arrhythmias than patients who did not develop arrhythmias (15.0% vs. 1.3%; *p* = 0.002). The HR for previous arrhythmias predicting future arrhythmias was 14.78 (95%CI = 4.29–50.82; *p* < 0.0001).

### 3.5. Global Pancreas T2* and Prediction of Arrhythmias

The low number of arrhythmias prevented carrying out a multivariate analysis including simultaneously all demographic, clinical, and MRI predictors. Therefore, several multivariable models adjusting the baseline pancreatic iron levels for different sets of significant variables in univariate analysis were generated (Table 3). Global pancreas T2* values remained an independent predictor for arrhythmias, also adjusting for age, sex, diabetes, left atrial area index, or history of previous arrhythmias.

In the model including both global pancreas T2* values and diabetes, only global pancreas T2* remained a significant independent predictor of arrhythmias.

### 3.6. Best Cut-Off of Global Pancreas T2* for Risk Prediction

**Figure 1:**
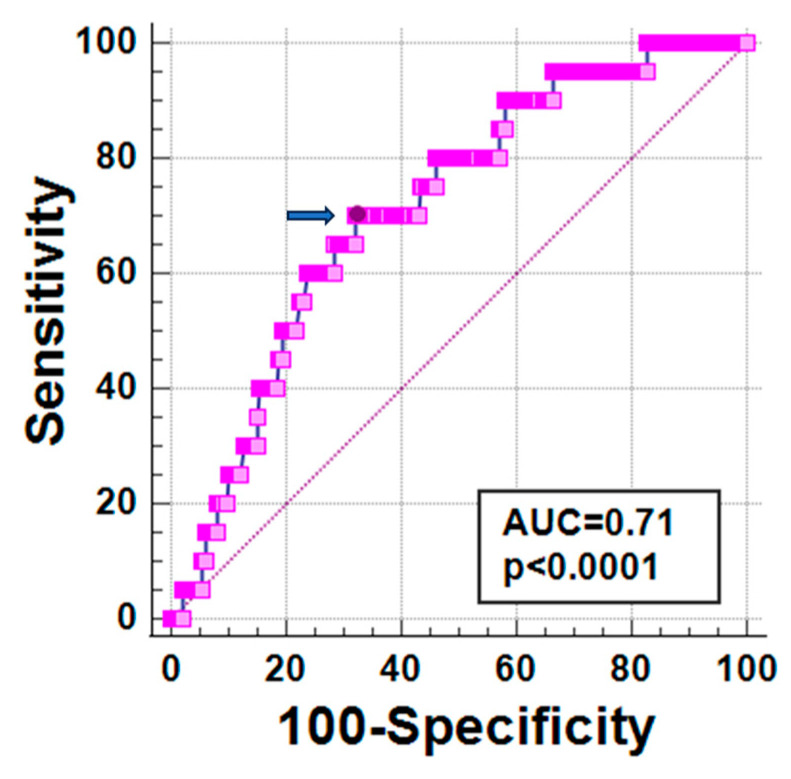
ROC curve analysis of global pancreas T2* values for new-onset arrhythmias. The optimal cut-off value of global pancreas T2* is indicated by an arrow.

At ROC curve analysis, a global pancreas T2* < 6.73 ms predicted the presence of future arrhythmias with a sensitivity of 70.0% and a specificity of 67.7% (*p* < 0.0001). The area under the curve was 0.71 (95% CI: 0.68–0.74) (Figure 1).

Global pancreas T2* values were dichotomized according to their median value (12.99 ms) and also according to the cut-off value found on ROC curve analysis.

**Figure 2:**
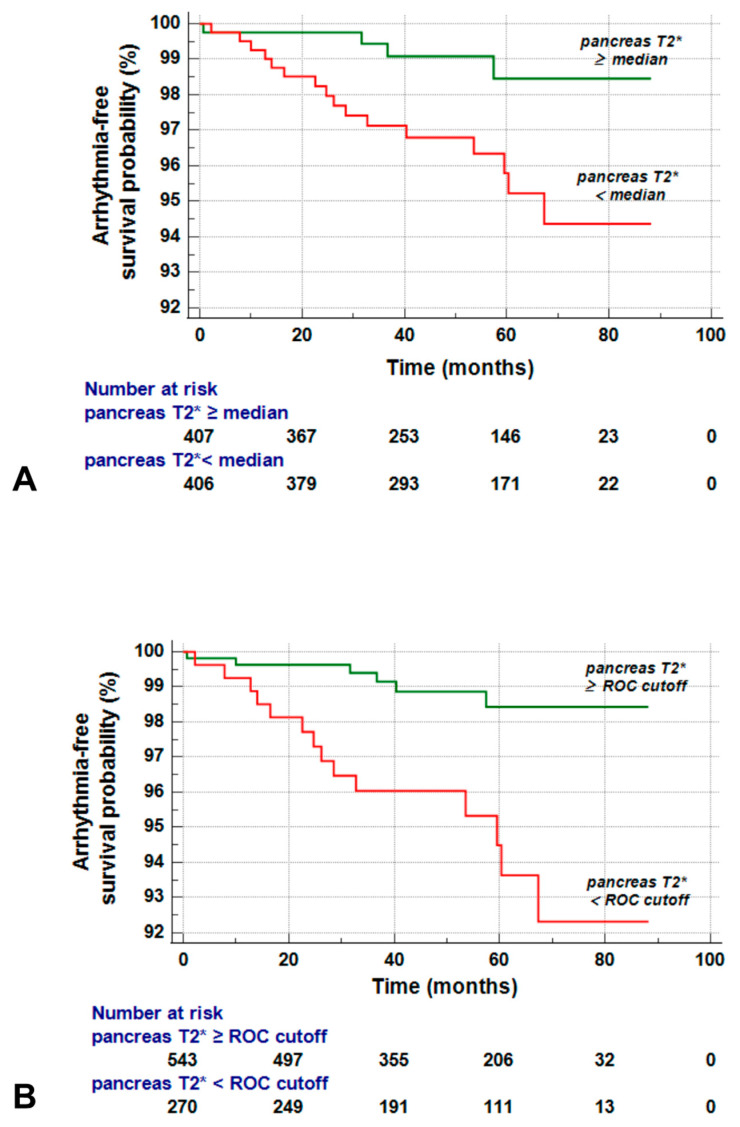
(**A**) Kaplan–Meier survival curves for global pancreas T2* values stratified below and above the median value against arrhythmias. (**B**) Kaplan–Meier survival curves for global pancreas T2* values stratified based on the optimal cut-off determined by the Youden index against arrhythmias.

Patients with a global pancreas T2* below the median level had an increased risk of arrhythmias compared to those with a global pancreas T2* above the median (HR = 3.69, 95% CI = 1.24–11.07; *p* = 0.019). Figure 2A shows the Kaplan–Meier curve. The log-rank test indicated a significant difference (*p* = 0.012).

When patients were dichotomized according to the optimal cut-off obtained from the ROC curve, the difference in the arrhythmia-free survival between the reduced and nonreduced pancreas T2* groups was slightly more pronounced (HR = 4.27, 95% CI = 1.64–11.14; *p* = 0.003). Figure 2B shows the Kaplan–Meier curve. The log-rank test demonstrated a significant difference (*p* = 0.001).

## 4. Discussion

Our study investigated the predictive value of pancreatic T2* measurements for cardiac complications in a large cohort of TDT patients regularly transfused since early childhood.

We took into account both HF-related and arrhythmia-related events. However, we had to focus our analysis only on arrhythmias, since only one patient developed HF during our follow-up. Left ventricular dysfunction and symptomatic heart failure typically arise in the later stages of cardiac siderosis, and there is potential for reversal with timely initiation of intensive iron chelation treatment [44,45,46]. The extremely low incidence of HF in our study may be explained by the fact that the majority of our patients were not naïve to cardiac T2* MRI and, during the MIOT project, the detection of myocardial siderosis had triggered an appropriate change or sustained escalation of chelation therapy [27]. Of note, the patient who developed heart failure showed pancreatic iron overload at the baseline MRI.

Our data confirm that supraventricular arrhythmias are the most common type of arrhythmia that cannot be predicted by the iron levels in the left ventricle of the heart [30]. Conversely, pancreatic T2* values emerged as a strong independent predictor of arrhythmias. This prospective association is well supported by the physiopathologic link between pancreatic iron overload and diabetes and the association between diabetes and cardiac arrhythmias. Indeed, in line with a previous study conducted on a smaller cohort of TM patients [30] and with meta-analyses of the general population [47,48], in our population, DM was significantly associated with an increased risk of developing arrhythmias. The connection between diabetes and arrhythmias involves a combination of different mechanisms. Diabetes can affect the autonomic nervous system, lead to structural changes in the heart’s atria and ventricles, and trigger various molecular changes in the heart, affecting ion channels and cellular signaling pathways involved in cardiac rhythm regulation [49,50]. Surprisingly, in a multivariate model including both diabetes and pancreas T2*, only pancreas T2* values emerged as a significant prognosticator of arrhythmias, suggesting the presence of other mechanisms involved in the link between pancreatic iron and arrhythmias. Iron deposition in the heart’s atria can have a significant impact on the occurrence and development of supraventricular arrhythmias. Abnormal iron levels can lead to dysregulation of calcium handling, affecting the normal electrical signals, and to changes in the expression and function of ion channels, which regulate the heart’s electrical activity, and promote oxidative stress, creating the electrical and structural remodeling that predisposes to supraventricular arrhythmias [51]. It has been proven that increased levels of reactive oxygen species (ROS) are associated with atrial fibrillation through changes in both local (atrial cardiomyocytes) and systemic (serum) activity of nicotinamide adenine dinucleotide phosphate oxidase (NOX), an enzyme complex that generates ROS [52,53,54]. The excess ROS in cardiomyocytes can cause oxidative damage to cellular components and disrupt normal cellular signaling pathways, contributing to the development and perpetuation of atrial fibrillation. Moreover, elevated levels of NOX can promote fibrosis by triggering the activation of transforming growth factor beta, which contributes to an increased susceptibility to atrial arrhythmias. Pancreatic iron overload was demonstrated to be significantly correlated with iron overload measured in the mid-ventricular septum [32,33,34] or the whole LV myocardium by a segmental approach [15]. This association was attributed to the presence of the same L-type Ca^2+^ channels (LTCC) taking up circulating NTBI [36]. These channels are highly expressed in both neonatal and adult atrial and ventricular myocytes. Therefore, it may be postulated that pancreatic iron deposition precedes atrial iron loading as well. Unfortunately, with the current noninvasive MRI techniques, the T2* measurement in the thin atrial wall is not robust and is affected by partial volume effects, preventing addressing this issue. Further studies are needed to confirm the prospective link between pancreatic iron and arrhythmias and to better elucidate the involved pathophysiological mechanisms.

The introduction of a T2* cut-off of 6.73 ms may help identify patients at high risk for arrhythmias. In these patients, developing targeted interventions to manage pancreatic iron overload may help prevent arrhythmias and improve patient outcomes. In this context, recent longitudinal studies demonstrated that the three iron chelators in monotherapy were equivalent in terms of reduction of pancreatic iron levels [55], emerged as a difficult and slow process, while combined deferiprone + desferrioxamine therapy was significantly more effective versus both deferiprone and deferasirox in monotherapies [56].

In addition to pancreas T2* values, increased left atrial area index emerged as a significant MRI predictor of arrhythmias. Left atrium dilatation is recognized as an early feature of cardiac remodeling in TDT [57], where the atria are subjected to prolonged or repetitive stretching due to various factors, including increased cardiac output. The stretched and remodeled atrial tissue provides a substrate for the development of electrical abnormalities, such as slowed conduction and micro-reentry circuits, which can contribute to the initiation and perpetuation of atrial fibrillation [58,59,60].

Beyond the MRI, age and male sex emerged as significant predictors of arrhythmias. Aging itself is associated with changes in the shape and size of the heart chambers, in the cardiac mechanical and electrical system, and in the heart’s energy production and utilization [61,62]. These natural alterations increase the predisposition to cardiac arrhythmias. Moreover, as patients live longer, their susceptibility to arrhythmogenesis is further increased by comorbidities, traditional and nontraditional risk factors accompanying the aging process, and increased exposure to iron overload and increased cardiac output. The association between male sex and arrhythmias is in agreement with previous studies and has been attributed to an increased sensitivity to chronic oxidative stress with a consequent reduced tolerance to iron toxicity compared to females, especially in the long term [28,63].

In conclusion, in TDT patients who have received regular transfusions since early childhood, elevated pancreatic iron levels have significant prognostic significance for arrhythmias. Therefore, regular monitoring of pancreatic iron levels may improve stratification of arrhythmia risk, and the development of targeted interventions to manage pancreatic iron burden, such as intensification or modification of iron chelation therapy, may help prevent or minimize the associated complications and further improve the prognosis of TDT patients.

## Figures and Tables

**Table 1:** Demographic, clinical and MRI data at the baseline MRI.

Variable	All Patients (N = 813)	Patients without Arrhythmias (N = 793)	Patients with Arrhythmias (N = 20)	*p*-Value
**Females, N (%)**	444 (54.6)	438 (55.2)	6 (30.0)	0.025
**Age (yrs)**	36.47 ± 10.71	36.27 ± 10.72	44.47 ± 6.81	<0.0001
**Splenectomy, N (%)**	449 (55.2)	435 (54.9)	14 (70)	0.179
**Pretransfusion hemoglobin (g/dL)**	9.67 ± 0.55	9.67 ± 0.55	9.68 ± 0.54	0.965
**Serum ferritin (ng/L)**	1061.24 ± 1330.85	1066.71 ± 1336.84	860.62 ± 1099.31	0.059
**Diabetes, N (%)**	89/796 (11.2)	83/776 (10.7)	6/20 (30.0)	0.007
**MRI LIC (mg/g/dw)**	5.88 ± 8.72	5.79 ± 8.52	9.02 ± 14.50	0.556
Global pancreas T2* (ms)	13.16 ± 10.49	13.32 ± 10.54	6.79 ± 5.11	0.001
Global heart T2* (ms)	37.32 ± 9.40	37.33 ± 9.38	36.77 ± 10.45	0.977
N. of segments with T2* < 20 ms	1.49 ± 4.06	1.49 ± 4.06	1.40 ± 3.89	0.792
**LV EDVI (mL/m^2^)**	82.15 ± 16.84	82.14 ± 16.67	82.59 ± 24.00	0.845
**LV EF (%)**	62.41 ± 6.59	62.38 ± 6.61	63.88 ± 5.43	0.335
**LV mass index (g/m^2^)**	54.81 ± 13.43	54.76 ± 13.44	57.18 ± 12.68	0.410
**RV EDVI (mL/m^2^)**	80.34 ± 17.63	80.34 ± 17.48	80.06 ± 23.97	0.436
**RV EF (%)**	60.86 ± 7.09	60.79 ± 7.09	62.59 ± 6.34	0.076
**Replacement myocardial fibrosis, N (%)**	56/224 (25.0)	54/218 (24.8)	2/6 (33.3)	0.633
**Left atrial area (cm^2^/m^2^)**	12.89 ± 2.50	12.82 ± 2.43	15.36 ± 3.69	0.002
**Right atrial area (cm^2^/m^2^)**	11.85 ± 2.38	11.79 ± 2.29	13.75 ± 3.95	0.014

N = number, MRI = magnetic resonance imaging, LIC = liver iron concentration, LV = left ventricular, EDVI = end-diastolic volume index, EF = ejection fraction, and RV = right ventricular.

**Table 2:** Results of univariate Cox regression analysis for the prediction of arrhythmias.

Independent Variable	Univariate Analysis	
	HR (95% CI)	*p*-Value
**Male gender**	2.71 (1.04–7.06)	0.041
**Age**	1.11 (1.05–1.17)	<0.0001
**Splenectomy**	1.75 (0.67–4.54)	0.254
**Pretransfusion hemoglobin**	1.12 (0.49–2.56)	0.780
**Serum ferritin**	1.00 (0.99–1.00)	0.549
**Diabetes**	3.15 (1.21–8.19)	0.019
**MRI LIC**	1.02 (0.99–1.05)	0.113
Global pancreas T2*	0.89 (0.81–0.98)	0.015
Global heart T2*	0.99 (0.95–1.04)	0.893
N. of segments with T2* < 20 ms	0.99 (0.88–1.11)	0.831
**LV EDVI**	1.00 (0.97–1.03)	0.915
**LV EF**	1.03 (0.96–1.11)	0.409
**LV mass index**	1.01 (0.98–1.05)	0.580
**RV EDVI**	0.99 (0.97–1.03)	0.870
**RV EF**	1.06 (0.99–1.13)	0.122
**Replacement myocardial fibrosis**	1.46 (0.27–7.99)	0.662
**Left atrial area index**	1.31 (1.16–1.49)	<0.0001
**Right atrial area index**	1.29 (1.12–1.45)	<0.0001

HR = hazard ratio, CI = confidence interval, MRI = magnetic resonance imaging, LIC = liver iron concentration, LV = left ventricular, EDVI = end-diastolic volume index, EF = ejection fraction, and RV = right ventricular.

**Table 3:** Prospective association between pancreatic iron levels and cardiac arrhythmias: multivariable-adjusted Cox regression analysis accounting for several confounding variables.

	Multivariate Analysis with Global Pancreas T2* as Fixed Independent Variable		
	Predictor	HR (95% CI)	*p*-Value
**Model 1: crude**	pancreas T2*	0.89 (0.81–0.98)	0.015
**Model 2: inclusion of age and gender**	pancreas T2*	0.88 (0.78–0.99)	0.026
	age	1.11 (1.05–1.19)	0.001
	male gender	3.48 (1.32–9.15)	0.012
**Model 3: inclusion of diabetes**	pancreas T2*	0.89 (0.82–0.98)	0.023
	diabetes	2.37 (0.91–6.21)	0.078
**Model 3: inclusion of left atrial area index**	pancreas T2*	0.89 (0.79–0.98)	0.024
	left atrial area index	1.39 (1.20–1.62)	<0.0001
**Model 4: inclusion of previous arrhythmias**	pancreas T2*	0.89 (0.82–0.98)	0.021
	previous arrhythmias	11.32 (3.27–39.19)	<0.0001

HR = hazard ratio, and CI = confidence interval.

## Data Availability

The data presented in this study are available on request from the corresponding author. The data are not publicly available due to privacy.
